# Caring for People with Severe Brain Injuries: Improving Health Care Professional Communication and Practice Through Online Learning

**DOI:** 10.1097/CEH.0000000000000486

**Published:** 2023-01-30

**Authors:** Julie Latchem-Hastings, Geraldine Latchem-Hastings, Jenny Kitzinger

**Affiliations:** **Dr. J. Latchem-Hastings:** Lecturer/HCRW Postdoctoral Fellow, School of Healthcare Sciences, College of Biomedical & Life Sciences, Cardiff University, Eastgate House, Cardiff, United Kingdom. **Dr. G. Latchem-Hastings:** Senior Lecturer, School of Healthcare Sciences, Cardiff University, Ty Dewi Sant, University Hospital of Wales, Heath Park, Cardiff, United Kingdom. **Prof. Kitzinger:** Professor of Communications, Journalism, Media and Cultural Studies, Cardiff University, Two Central Square, Central Square, Cardiff, United Kingdom.

**Keywords:** vegetative state, brain injury, rehabilitation, continuing professional development, e-learning, online teaching, virtual learning

## Abstract

**Introduction:**

Severe brain injuries can leave people in prolonged disorder of consciousness resulting in multifaceted medical, nursing, and rehabilitative needs that can be challenging for even the most experienced multidisciplinary team. The complexities of care, communication with families, and best interest decision-making about medical interventions means there is a need for ongoing training in clinical, social, ethical, and legal aspects.

**Methods:**

Using a combination of group discussions, interviews, and questionnaires with learners, this article reports an evaluation of designing and delivering an interprofessional, online work-based course to health care professionals caring for prolonged disorder of consciousness patients.

**Results:**

There were challenges for staff uptake because of COVID-19, but engaging with it increased knowledge in defining and diagnosing patients' conditions, understanding multidisciplinary team roles, communicating with families, and navigating legal and ethical issues. Course participation also enhanced critical and reflective thinking skills, provided a sense of connection to other professionals, and generated plans to improve service provision.

**Discussion:**

Online learning that enables health care professionals to engage at their own pace and also come together as an interprofessional community can provide invaluable continuing professional development and help to enhance joined up, holistic patient care. However, achieving this requires significant investment in creating research-led, multimedia, learning materials, and courses that include synchronous and asynchronous delivery to combine flexible study with the opportunity for peer networks to form. It also depends on a commitment from organizations to support staff online continuing professional development.

In response to the COVID-19 pandemic, most forms of continuing professional development (CPD) for health care professionals (HCPs) had to move rapidly online. Since then, National Health Service (NHS) and health education strategies^1^ have prompted further strategic thinking about how to move beyond emergency online delivery to a more sustainable and positive engagement with the potential of online CPD. This article evaluates a multimedia online course designed to develop HCPs' skills in caring for patients with prolonged disorders of consciousness (PDoC).

Prolonged disorders of consciousness is an umbrella term for three conditions—coma, vegetative, and minimally conscious states. These conditions, caused by severe brain injury, are associated with profound motor, cognitive, sensory, and functional deficits that require full and continuous care.^2^ The complexity of such patients' needs is complicated further by social, ethical, and legal contexts. Our own research has highlighted urgent training requirements for HCPs including clarification around diagnosis, improving communication with families, and enhancing decision-making about life-sustaining interventions.^3–7^

In a bid to address these learning needs, we (members of the Coma and Disorders of Consciousness Research Centre) had already delivered in-person talks and training days to over 5000 practicing HCPs since our formation as a research center in 2010 (see list at: www.cdoc.org.uk). In 2018, we started to develop an online learning course designed to be delivered over a ten-week period for interprofessional CPD in workplace settings, combining online interactive self-study modules with “real-time” virtual seminars and tweet chats. The course consists of three learning sets, each involving two or three modules (Table 1); each set involves between four and 8 hours of independent study, depending on the learner's knowledge base and the depth to which they wish to explore each area.

**TABLE 1.** Course Overview

Learning Set	Modules
1: Introduction to PDoC care (Estimated: 4–8 hours of study)	• Definitions and diagnosis • Core practices • MDT working
2. Communicating with families (Estimated: 4–8 hours of study)	• Family experiences • Family views of therapies
3. Law and ethics (Estimated 4–8 hours of study)	• Best interests and the law • Treatment Disputes

## The Existing Literature About Online Learning in Health Care

Learning through online resources has been part of CPD for HCPs for over 20 years. Initially held up as a mechanism for educational delivery that met the needs of the “Modernization Agenda”,^8^ online CPD was considered to help staff motivation and encourage practitioners to take responsibility for personal development in evidence-based practice.^9,10^ Initially, most commonly used as a way to test and evidence compliance with key governance requirements, online learning subsequently developed significantly in pedagogical sophistication. It is now being successfully used to support rehabilitation professionals to, for example, learn core practice knowledge and skills from human anatomy^11^ to dysphagia competencies,^12^ manual therapy techniques in physiotherapy^13^ and stroke assessment.^14^

The various pros and cons to workplace online learning in health care have been extensively discussed.^15,16^ The ability of this pedagogical medium to change the way in which interprofessional groups and teams work and learn together has been particularly noted. One review found that e-learning technologies such as interactive menus, online case studies, and video clips promoted interprofessional interaction and improved the quality of collaborative learning, which led to improved team decision-making skills.^15^ Online learning also provides an environment within which a collaborative community can be fostered and it facilitates opportunities to train as whole teams, a benefit welcomed by team leaders.^15^ Additional advantages include: more flexibility and time-effective access to learning opportunities^17^ and the ability to deliver standardized education for teams spread across multiple clinical sites^18–20^ and different areas of the country.^21,22^

However, online learning does not come without its challenges: some learners can feel isolated^23^; there can be issues about access to necessary hardware and software^15,24^; too many courses are poor-quality and there has been a lack of acknowledgment of, or investment in, the true cost of developing good online materials.^25^

Despite the challenges, overall, online learning for HCPs, particularly those in multidisciplinary teams, is now widely recognized as important. It was this focus on tapping into the positive aspects of online learning—particularly enabling interprofessional education across geographic regions and health care settings—that shaped the decision to go online and the development of the “Caring for PDoC patients” course.

## The Development, Design, and Delivery of Our Online Course

### Course Delivery

Our ten-week course covering the full gambit of PDoC care was offered using a “flipped classroom” approach—providing “real-time” learning opportunities alongside asynchronous learning materials. Our inclusion of synchronous gatherings reflected the fact that work-based online CPD, which is undertaken with colleagues can enhance learning through the discussion of lived real-life situations and offer opportunities for immediate direct application of new knowledge.^26,27^ In planning how to deliver the course, we also sought to create a learning environment where HCPs from differing professions could connect across teams and organizations to broaden the learning environment beyond a single workplace and to increase understanding and communication across the different settings through which PDOC patients may move (eg, from hospital to rehabilitation). Dialogue with tutors, and, crucially between learners, was carried through in real-time synchronous discussion in the regular tweet chats and seminars—all important ways of creating a “community of inquiry and practice” among learners themselves.^28–30^

### Course Development

The course was built by a team from the Coma and Disorders of Consciousness Research Centre. The team had extensive experience of HCP education, and in relevant academic research including looking at family perceptions of therapy,^3^ decision-making around life-sustaining treatment,^6^ and clinical practice within multidisciplinary neuro-rehabilitative teams. We also had previous experience of building an online multimedia Healthtalk resource for families of PDoC patients (https://healthtalk.org/family-experiences-vegetative-and-minimally-conscious-states/overview). This meant we had relevant skills and were aware of the potential reach and impact of such online resources.

The core team benefitted from excellent technical support from Neil Pollock, Learning technologist at Cardiff University, and Liz Fahy, WordPress Developer at GeckoSurfing. End user involvement from health care professionals was key from the outset and informed iterative development, in keeping with the literature, which highlights that “co-production” underpins positive learning experiences.^31,32^ We also engaged directly with families of PDoC patients in developing the course for example, inviting them to provide feedback on different sections. In addition, two members of the core course-development team had personal experience of having had a relative in a vegetative/minimally conscious state.

### Course Design

Alongside providing key updates on legal and practice guidelines, the course was designed to promote multidisciplinary working, reflective learning, and critical thinking. To this end, we often juxtaposed clips from interviews that highlighted contrasting perspectives (eg, different points of view from family and multiple health care professions). We also introduced perspectives from a broader range of disciplinarily lenses (eg, media and cultural studies, the arts and sociology). This meant the course presented new ways of looking at how care for this patient population is constructed and the broader influences on how medicine is organized and enacted. Such exploration ranged from the social construction of medical diagnosis^33^ to debates about the role of modern medicine.

We were also informed by the literature on designing online courses, which highlights the importance of clear structure and learning objectives, providing content in a variety of forms, and offering opportunities for instant feedback and for in-depth reflection.^34^

The course is multimedia incorporating a mix of text (rarely more than a few paragraphs at a time) with videos (mostly under 5 minutes), where learners can watch the teaching team present core information or see clips from interviews with family members, clinical and legal experts, and frontline staff from across health care professions (eg, Occupational Therapy, Nursing, Physiotherapy etc). Other media used includes newspaper clippings, court transcripts, and art, poetry, and shadow puppet theatre (specific artistic output developed from our research with PDoC families).

Throughout the course, a variety of tasks are encouraged to tap into different ways of engaging with new knowledge and ideas (eg, see Laurillard's^35^ discussion of the effect of assigning “productive”, “assimilative”, and “communicative” exercises). Interactive elements include questions to consider while listening to a particular interview clip, quizzes to allow self-testing about key facts, and multiple choice options designed to help students to contextualize and think through what they are learning.^36^ Alongside this, learners are asked to write reflective practice pieces throughout the course.

The literature about online teaching also highlights the value of conveying the persona of course tutors.^34^ Brief profiles were therefore presented of each of the four people central to course development and we also created avatars of ourselves that pop up regularly to guide learners through the course and are sometimes placed in dialogue with one another for example, in “water cooler” moments with speech bubbles capturing differences of perspective or unpicking a particular point of view.

## EVALUATION METHODS AND COURSE OUTCOMES

This next section examines course outcomes. Following Levels 1 to 4 of Moore's Outcomes framework,^37^ we provide an overview of student participation and responses to the course and present data on changes in knowledge and perspectives and the plans for action developed by learners as a result of participation. The course assessment was not however designed to assess clinical performance or observed competency within the workplace and therefore levels 5 to 7 of the framework are not applicable here.

In total, 222 people registered for the course that was run in 2019 and 2020 (their profession and locality can be found in Table 2 and Table 3). These professionals came from 65 organizations (across the NHS, independent and voluntary sectors) and worked in intensive care, acute and general wards, rehabilitation community, and long-term care settings. We attracted learners from a range of geographical locations across the UK and the Republic of Ireland and from a range of sites: from large city teaching hospitals to rural county hospitals, community services and integrated acute and community services on individual islands such as the Channel Island, Jersey. Some joined as individuals, whereas many joined as full multidisciplinary teams.

**TABLE 2.** Applicant Demographics

Profession:	Number of Applicants:
Occupational Therapist	53
Physiotherapist	49
Nurse	40
Speech and Language Therapist	28
Psychologist	20
Doctors	6
Care assistant	6
Case manager	6
Dietitian	1
Therapy assistants	5
Other	8
**Total**	**222**

**TABLE 3.** Applicant Demographics–Country

Country/Nation:	Number of Applicants:
England	176
Wales	20
Republic of Ireland	16
Jersey	8
Northern Ireland	1
Scotland	1
Total	222

Our evaluation data consists of group discussion with participants at four sites (conducted in 2019 when face-to-face contact was still possible), seven audio-recorded one-to-one interviews conducted via zoom (in 2020), and 66 end-of-course questionnaires including participants self-rating of their knowledge (from both the 2019 and 2020 cohort). We also draw on our observation notes taken during live seminars and analysis of tweet chats.

### Ethics

This evaluation was considered exempt from ethics approval by the chair of the School of Journalism, Media and Cultural Studies ethics committee, Cardiff University. We however note here considerations made around privacy and confidentiality in relation to our seminars and tweet chats. Learners were reminded not to give identifiable information about patients or other team members not on the course. Seminars were recorded, but only for sharing with others registered on the course—and were made available for a limited time. Tweet chats were conducted in accordance with core guidelines and policies for ensuring professional practice standards are upheld within these spaces.^38,39^

### Participation

Of the 222 people registered for the course, 165 (ie, 74%) started on the course and completed at least the first learning set. However, only 66 people (40% per cent of those who started) completed all three learning sets and filled in the final feedback questionnaire. It is difficult to know how to interpret “whole course completion’ data given the impact of COVID-19 at the time and given the ambitiousness of combining three learning sets into one time-intensive ten-week course—something we changed subsequently where each learning set was delivered on its own over three to four weeks each, as a distinct learning commitment. For comparison, in 2022, we offered a course called “Introducing PDoC” (https://cdoctraining.org.uk/). This was based on a development of just the first learning set of our original mega course; 208 students registered for this, 68% of those started it, and 81% of those managed to complete it.

Learners who were able to maintain participation in 2019 and 2020 and engage with the real-time seminars were enthusiastic and discussion included, for example, directing each other to diverse resources, sharing how difficult it could be to feel the target of family anger; or reflecting on their own moral dilemmas in PDoC care. As one Speech and Language Therapist commented: “It has reassured me that patients in PDoC are extremely challenging to work with and that it’s ok to be not ok with this sometimes” (R27). Discussion also addressed differences between learners—for example, staff at some specialist centers challenged colleagues working in acute settings by highlighting the dangers of promoting unreasonably high expectations of what specialist rehabilitation may achieve.

The evening Twitter/Tweet chats also had high levels of engagement. For example, during the first evening, there were 742 tweets from 67 users with links offered to 23 items (research papers, professional standards and policy documents). What was exciting from an education perspective was the fact that the “chats” continued for hours after the formal one-hour twitter chat ended and there was a trend of ongoing engagement each week until the next scheduled Twitter chat when activity spiked once again. The first Twitter chat recorded 934.8K Twitter impressions reflecting the fact that there was also significant attention from people outside of the course.

### Satisfaction

Learners praised the clarity of the structure, bite-size learning units, and diversity of course materials and several highlighted their comprehensive nature: **“**Thank you for collaborating and creating this. It’s 15 years of my experience taught in a few weeks” (Physio, R20). Different learners highlighted the value of different aspects of the course: ranging from the stretching theoretical or ethical discussion right through to practical tips: “Little snippets of advice on clinical practice were valuable—I'd be reading it in the morning and going in and putting it into practice that very day” (Clinical Psychologist, Int 5).

Learners commented positively on the “interactive and dynamic” elements (Nurse, R59). A clinical psychologist, for example, wrote: “I liked the videos, it made it very ‘alive’, it was like listening to a colleague. I also liked the reflective pieces. It allowed to be more active and present” (R36). The variety of perspectives illustrated were appreciated as “invaluable to the dialogue” (Occupational Therapist R16) and supporting open conversation, “The course examined so much, the good, the bad, the ugly, the difficult—it felt like a safe place to explore” (R16).

Learners also highlighted how much they enjoyed being part of an interdisciplinary learning cohort: “Heartfelt thanks, it has really made feel as we're not alone” (Occupational Therapist, R50); “It was great to hear and benefit from other groups of professionals from different centers and hear from their experiences, a lovely way to learn” (Physio, R36).

### Declarative and Procedural Knowledge Gains

The first learning set, “*Introducing PDoC Care”*, was predominantly designed to establish common core understandings. The module on “Definitions and Diagnosis” within this learning set, for example, helped those new to PDoC to understand the difference between vegetative and minimally conscious states and provided an introduction to the potential meaning (or not) of different behaviors from the patient. This learning set increased health care professionals' confidence about assessing patients' levels of consciousness—shifting from 3.7 to 4.5 on a five-point Likert scale—with one respondent noting that she had learned that her original confidence in her knowledge was actually misplaced. The other two modules within this learning set (“Core practices” and “MDT working”) helped those new to PDoC to gain an overview of basic care and increased knowledge about the diverse roles within the multidisciplinary team (from 2.5 to three on a three-point scale).

The second learning set, *‘Communicating with Families’,* increased appreciation of different family perspectives and increased confidence communicating with families (from 1.7 to 2.3 on a three point scale). Learners made comments such as: “I understand more about what families may be experiencing and I want to listen to them more” (Physiotherapist R7). They also highlighted specific learning such as: “how family perception changes from acute to long term and how to change my expectations and vocabulary, how to provide them with support for the long-term not just for the now” (Physiotherapist, R45). Particular interest was expressed in the insights provided by Latchem et al^3^'s analysis of family interpretations of therapies, which showed how families may misunderstand what therapies can deliver.

The third learning set, *“Law and Ethics”,* increased understanding of law and ethics (from 3.7 to 4.2 on a five-point scale). Learners appreciated being brought up-do-date with the guidelines from the British Medical Association and Royal College of Physicians published in 2018 and 2020.^40,41^ Some said they had believed they knew the law and were shocked, but valued, discovering gaps in their understandings. Many reported a positive increase in confidence in this area, including in relation to having discussions with families about what the person would have wanted. This was evident across the full spectrum of professions (eg, Nurse R66, Speech and Language Therapist R39, Occupational Therapist R44, Physiotherapist R7). In particular, learners came away with a practical focus on how to get information about, and give weight to, the patient's wishes in the decision-making process (in accordance with the Mental Capacity Act 2005). This was identified by many as the single most significant take-away message from the entire initiative and a significant shift in how they saw decision-making.

### Competence (Demonstrating Intent to Change and Putting Learning into Practice)

Learners demonstrated how they were able to challenge aspects of their previous practice. For example, one specialist occupational therapist reflected on how she and her colleagues had come to recognize how a preoccupation with diagnosis in isolation could be an obstacle to patient-centered care.

“Before we’d got a bit sucked into the assessment and the diagnosis being the be all and end all: “Is this person in the vegetative state? Are they minimally conscious? And what level?” I think the course really helped us as a collective think a bit broader in terms of let us not get too kind of drawn into where on that continuum of consciousness that individual is but more [focus on]: What does everyday life mean for that person and where does that kind of leave the “So what?” […] It all came together as we were putting work in place to review the structures that we have. It also gave us that reflective philosophical knowledge base as well as the theory to help us flesh out that framework.” (Occupation Therapist, interview no 6) (View the film clip of this reflection here: https://youtu.be/vrECt7c6g2Q)

The “*Law and Ethics”* learning set also particularly helped staff think critically about making judgements about what was right for patients: “I am now way more conscious of my own values and beliefs and their impact” (Occupational Therapists R44); “I will try and be more impartial and not to impose own opinions” (Nurse R23); “[I will remember] to keep the patient at the center of all we do and…not to treat at all costs just because we can” (Consultant Clinical Neuropsychologist R27). One consultant we interviewed reported profound ways in which her thinking had been affected by the course:

“I might be acting in line with my own ingrained ethical and moral concepts—but that might not be in the best interests of that particular patient. I need to understand my limitations.” (Rehabilitation Consultant, int. 7).

An action plan from this consultant included now being sure she put clinically-assisted nutrition and hydration on the agenda for discussion and ensure that decisions about life-sustaining treatment are regularly reviewed (in line with the RCP 2020 guidance).

One of our core aims was to stimulate improvements to practice (and to capture intent to change). The impact of the course in relation to such aims was particularly clear in the learning set around law and ethics (as illustrated by the consultant quoted above) and was also evident in the discussion about communicating with families. For example, learners talked about a determination to be more “honest” with families about prognosis. One Speech and Language Therapist wrote “as a result of this course, [I will] be a bit braver with families and honest with them about prognostic guesses'” (R6). In PDoC care, allied health professionals play a significant role in assessing the level of consciousness of PDoC patients (and diagnostic category). Their regular communication with families often requires them to discuss both current diagnosis and potential long-term outcomes. These can however be clinically difficult to predict with accuracy, particularly early on after injury, although it is often possible to identify a spectrum of possible recovery and this can become narrower given time and systematic and skilled assessment. In group discussions, MDT members reported that they would now “have courage to have those discussions” and discussed their commitment to putting in place “robust systems…to reduce the impact of professional biases and to ensure the correct route is followed by all” (Group discussion, Site C). Actions included using tools from the course:

“The most important things I learnt on this course and will use in my work are.... that final chart of what can go wrong in establishing best interest decisions from Derek Wade [a neuro-rehabilitation consultant who contributed to the course]—very helpful as guidance for how not to do it” (Speech and Language Therapist, R56).

Learners also reported drawing on what they had learned from the course, and from each other, to push for improvements in their own workplaces:

“knowing the other units are more proactive in having Best Interests discussions, we have more ammo to go back to our consultants with! The most important thing I learned on this course was to start the Best Interests process sooner” (Physiotherapist, R12).

A great deal of thought had been put into how to improve practice (or address poor practice):

“We are at risk of failing to assess and review decisions that were made in relation to some of our patients [and I've learned] how important it is to initiate the discussion with families from the very beginning. I find the infographic from Dr Wade a good prompt...It could even be part of the patient's file and be brought out at the team meeting so we can check the various steps and keep monitoring.” (Psychologist R35).

In summary, HCPs engaged extensively with the course (within the limitations imposed by COVID-9), tackling some complex clinical, social, ethical, and legal issues with enthusiasm and commitment. Overall, taking the course led to improvements in knowledge, understanding and confidence, provoked reflection, enhanced critical thinking, and provided tools to help create practice change.

## DISCUSSION/CONCLUSION

The course evaluation illustrates that multimedia, online training materials can provide an engaging and accessible learning experience to improve understanding of a clinical condition and core care practices, and enhance communication with families and decision-making for patients. However, risks and opportunities need to be considered and may be particularly important with the mass move online prompted by COVID-19.

First, the challenges. Although COVID-19 stimulated a huge update in the use of online conferencing platforms for private social use and to enhance access to health care, NHS organizations were far from ready for such a surge in technological usage. When we first launched the course, many HCPs who signed up did not have access to a PC or laptop in their clinical areas that had speakers, microphone, and camera; also the bandwidth required to enable continuous streaming of online conferencing platforms was often lacking. We had to go to significant lengths to support access by, for example, communicating with Trust Information Technology staff to ensure the removal of blocks to conferencing systems and even sending missing pieces of equipment such as webcams to those unable to get in-house support. It is likely that the pandemic has forced a rapid investment in updating technology. One thing that is unlikely to have changed, however, is the pressure on staff time. Although we had asked learners to seek support from their managers to provide protected learning time, service needs will always trump CPD. It was therefore important that learners who had to miss seminars because of clinical needs were provided with opportunities for alternative engagement. Our setting up of evening twitter chats formed part of this remedy along with the recording of seminars and the sharing of these recordings with those unable to attend in real-time.

Moving on to the risks in/to developing online courses for HCPs, we would highlight that quality does not come simply by reproducing classwork plans online—but from the skilled and often labor-intensive (and therefore costly) creation of engaging content. In the rush to deliver online learning under “emergency conditions,” there has been a danger of underestimating the skills and time needed^42^—putting huge pressure on staff developing such resources and also exposing students to poor-quality online materials that can cause disengagement with online formats in their entirety.

Despite the challenges and the risks to education moving online, the opportunities blended online learning offer to HCPs is extensive and has a place during and after a pandemic. Although the pandemic took workloads to an all-time high, even in so-called “post-pandemic” times, HCPs will remain as time pressurized as ever. The flexibility and accessibility online-learning formats offer will remain important.

Critically however, online learning that combines synchronous and asynchronous learning makes possible extensive benefits. For example, it provides opportunities to bring together HCPs of differing levels of experience and different parts of the health care system. The asynchronous components allow learners to be suitably prepared, with enough knowledge to enable their engagement with others in real-time. In the case of our course, this meant that therapy assistants for example could interact with clinical specialists over issues that affected everyone's practice and HCPs from large geographical areas could meet and learn together, across service and organization type. Although national and international conferences also enable such cross-country and organization meetings, their cost and the “time out” required in one go, can be a barrier to attendance. Furthermore, opportunities for purely PDoC focused events (or any other “minority” condition) can be limited in comparison to more prevalent neurologic conditions such as stroke and multiple sclerosis.

To conclude, providing quality-blended online learning for HCPs requires significant investment. However, the learning outcomes achieved and the opportunities it offers in engaging and bringing together diverse HCPs creates a pathway toward developing significant changes to clinical practice—which in turn, could improve the care and treatment of patients. Follow-up with our learners in future will explore the extent to which planned changes have been delivered and reported changes have been sustained and built upon. In the meantime, we have revised the initiative to respond to suggestions for improvements from our learners and are rerunning course(s) in 2023; we have also obtained funding to develop new education materials: immersive simulations of best interest meetings to refine dynamic skills in clinical and best interests decision-making.

**Lessons for Practice**

■ Multimedia, online continuing professional development can provide an engaging learning experience to improve health care professionals' understanding of complex clinical conditions, communication with families, and best interests decision-making
■ Online continuing professional development may be particularly useful for learning about rarer conditions and addressing situations where expertise may be largely concentrated in specialist centers and connecting remotely can enhance cross region and country multidisciplinary contact.
■ To maximize learning in this format of continuing professional development, there is the need to invest in research led, high-quality interactive online materials alongside supporting technological access requirements and providing staff training time.
